# The Associations of Peer-Rated Popularity and Likeability With Dark Triad Personality Traits in Adolescent Groups

**DOI:** 10.5964/ejop.11667

**Published:** 2024-08-30

**Authors:** Zsolt Péter Szabó, Natália Zsuzsanna Orosz, Réka Gulyás, András Láng

**Affiliations:** 1 Institute of Strategy and Management, Corvinus University of Budapest, Budapest, Hungary; 2 Department of Social Psychology, Faculty of Education and Psychology, ELTE Eötvös Loránd University, Budapest, Hungary; 3 Institute of Psychology, Faculty of Humanities and Social Sciences, University of Pécs, Pécs, Hungary; Victoria University of Wellington, Wellington, New Zealand

**Keywords:** Dark Triad personality traits, Machiavellianism, narcissism, psychopathy, social acceptance, likeability, popularity

## Abstract

One of the most significant challenges in adolescence is the pursuit of social acceptance, which can manifest in various forms, including likeability and popularity. Achieving social acceptance is associated with positive outcomes, while its absence is linked to adverse consequences. Existing research into the personality determinants impacting the ability to elicit likeability or gain popularity remains limited, primarily focusing on the influence of Big Five traits. This study aimed to explore the relationships between self-reported Dark Triad traits -encompassing Machiavellianism, subclinical psychopathy, and subclinical narcissism- and peer-rated likeability and popularity in a naturalistic high school classroom setting. The sample comprised 184 secondary students (98 females, 86 males) with an average age of 16.29 (*SD* = 1.36). Participants self-reported their Dark Triad traits and provided peer ratings through sociometric questions related to likeability and popularity. Our findings indicated that narcissism was significantly and positively associated with both likeability and popularity. In contrast, psychopathy and Machiavellianism exhibited minimal associations with measures of social acceptance. We discuss the theoretical and practical implications of these findings.

One of the prominent challenges during adolescence is the pursuit of social acceptance, which can take on different forms, including likeability and popularity. Achieving social acceptance is linked to positive outcomes, such as improved mental health, happiness, and self-esteem (e.g., De Bruyn & Van Den Boom, 2005; Harris & Vazire, 2016). Conversely, its absence is associated with negative outcomes like loneliness (Zhang et al., 2014) and may even impact academic performance (Guinouard & Rychlak, 1962).

Understanding the determinants of social acceptance, especially during adolescence, is essential for promoting overall well-being and psychological development. The significance of personality traits was recognized early within the literature (Guinouard & Rychlak, 1962). The notion that dispositions such as selfishness, low empathy, limited agreeableness, and reduced honesty-humility, which are associated with Dark Triad personality traits (henceforth DT), might hold predictive power seems intuitively plausible. However, the existing body of literature addressing this issue is notably constrained in scope.

The investigation into the relationship between DT and social acceptance primarily focuses on their correlation with mate choice and romantic relationships (Jonason & Schmitt, 2012). This study aims to determine whether an association exists between elevated levels of DT among secondary school students and the likelihood of gaining popularity and/or likeability within the classroom.

## Literature Background: Likeability and Popularity in Adolescent Groups: Relationships With Personality Traits

De Vries et al. (2020) argue that fulfilling the need to belong (i.e., social acceptance) presents a significant adaptive developmental challenge, which is tackled through two pathways: likeability and popularity. Likeability is defined as the extent to which peers like and accept an individual (Hubers et al., 2016), and it correlates with prosocial behavior and low aggression (van der Linden et al., 2010). Popularity refers to the extent to which an individual is viewed as socially dominant and visible within a group (Hubers et al., 2016). Popularity signifies the level of prestige and influence an individual holds within the group. van der Linden et al. (2010) assert that popularity is a multifaceted construct, encompassing favorable attributes such as intelligence, friendliness, and attractiveness, as well as less favorable traits like aggression, arrogance, and manipulativeness.

The concept of social acceptance as a finite resource, wherein likeability and popularity are viewed as scarce commodities (de Vries et al., 2020), has evolutionary roots. Acquiring peer relationships through likeability or popularity served as a valuable asset in evolutionary contexts (de Vries et al., 2020). It enabled individuals to establish bonds with peers to build a support network against threats. Furthermore, it facilitated the formation of relationships with the opposite sex for procreation.

The adaptive challenges rooted in evolutionary history no longer exert the same influence within the contemporary milieu as in their original context. However, the increased need for peer belongingness remains evident during adolescence (de Vries et al., 2020). In late adolescence, the potential pathways for satisfying these needs diverge: individuals who are popular begin to exhibit different behavior from those who are liked (Cillessen & Borch, 2006). While a strong correlation exists between likeability and popularity during early adolescence, this lessens in late adolescence. Being liked becomes less crucial for attaining popularity, which becomes more closely associated with the ability to ‘get ahead’ (de Vries et al., 2020). The prevailing consensus in the literature is that, despite their commonalities, likeability and popularity are distinct concepts (Wolters et al., 2014).

Existing research, relatively limited in scope, primarily focuses on the impact of the Big Five personality traits in exploring the personality determinants influence individuals’ ability to elicit likeability or gain popularity (Harris & Vazire, 2016).

Regarding likeability, extraversion, agreeableness, and emotional stability have emerged as dispositional determinants (de Vries et al., 2020; Harris & Vazire, 2016; Hubers et al., 2016; van der Linden et al., 2010). However, the role of extraversion remains somewhat ambiguous. While communal aspects of extraversion, like sociability, display a clear link with likeability, the relationship is less evident for agentic aspects of extraversion, such as dominance (Harris & Vazire, 2016).

The most consistent predictor of popularity is extraversion, while agreeableness does not significantly predict it, further hinting at the conceptual differences between popularity and likeability (Anderson et al., 2001; de Vries et al., 2020; Hubers et al., 2016; van der Linden et al., 2010).

De Vries et al. (2020) examined the relationships of the HEXACO personality model with likeability and popularity. In contrast to the Big Five, HEXACO incorporates a sixth dimension, honesty-humility, characterized by traits such as sincerity, fairness, and modesty. Their study revealed a notable correlation in early adolescence—an inverse link between honesty-humility and popularity. This suggests that individuals with higher levels of honesty-humility are less likely to achieve popularity, reinforcing the notion that popularity has a dark, antisocial aspect (LaFontana & Cillessen, 2002; Sandstrom & Cillessen, 2006; Wolters et al., 2014). These findings, along with similar observations (Cheng et al., 2010; Rauthmann & Kolar, 2013; Zeigler-Hill et al., 2014) emphasize the value of comprehensively exploring the interplay between popularity, likeability, and DT to enhance our understanding of social acceptance in adolescent groups.

## The Dark Triad and Social Acceptance

The DT comprises three socially undesirable personality traits: Machiavellianism, subclinical narcissism, and subclinical psychopathy (Paulhus & Williams, 2002). These traits share a common core characterized by malevolent and exploitative behavior, low willingness to cooperate, low honesty-humility, and a propensity towards callousness and aggression (Kowalski et al., 2021). Despite these commonalities, significant differences exist among the three traits. These differences may be closely linked to variations in how individuals high in each trait are perceived by their peers.

Subclinical narcissism, particularly the agentic and grandiose types measured by the Short Dark Triad (Jones & Paulhus, 2014), is defined by grandiosity, entitlement, superiority, and dominance (Paulhus & Williams, 2002). Individuals high in grandiose subclinical narcissism typically display reactive aggression in response to ego-threatening situations. Subclinical psychopathy is marked by indifference toward social norms, impulsivity, hostile and instrumental aggressive behaviors, risk-taking tendencies, and low empathy (Truhan et al., 2021). Unlike the other two traits, Machiavellianism is characterized by its long-term, strategic, and flexible adaptation to changes in the social environment, as well as low levels of aggression (Bereczkei, 2018).

A notable research gap exists in understanding how individuals with DT are perceived and accepted in close-knit groups. Moreover, even the limited existing studies typically rely on self-report data in non-naturalistic settings, potentially leading to invalid conclusions (Muris et al., 2017).

While the negative aspects of grandiose subclinical narcissism undoubtedly present challenges within most long-term social groups (Prusik & Szulawski, 2019), some argue that its positive traits, coupled with its associations with extraversion (Szabó, Czibor et al., 2023; Szabó, Diller et al., 2023), can make individuals with high grandiose narcissism likable and popular. Recent research establishes connections between narcissism and attributes such as mental toughness and resilience (Papageorgiou et al., 2019; Szabó et al., 2022), enhancing their potential for advancement and visibility. Studies demonstrate that narcissistic traits often contribute to success (Rauthmann & Kolar, 2013) and leadership roles (Paunonen et al., 2006).

Research focusing on narcissism’s impact on forming and maintaining friendships, an aspect tightly linked to likeability (Laursen et al., 2023), reveals that individuals with elevated narcissism levels establish friendships for various reasons, including factors that establish the foundation for enduring relationships, like shared interests (Jonason & Schmitt, 2012). However, those with narcissistic traits exhibit reduced friendship commitment (Sauls & Zeigler-Hill, 2020), and narcissism is also linked to challenges in forging and maintaining relationships (Wehner & Ziegler, 2023). In naturalistic settings, their evaluations by others tend to be neutral (Rauthmann, 2012). Reactive aggression, also tied to narcissism, negatively predicts popularity (Stoltz et al., 2016).

High psychopathy scores are associated with confrontational behavior, hostile and instrumental aggression, and bullying (Boddy, 2010). The ability of individuals with high psychopathy scores to attain high status and popularity in adult groups remains a subject of considerable debate. While a substantial body of research suggests that they can advance through aggressive, risk-taking, and manipulative behaviors (e.g., Babiak et al., 2010), recent findings challenge this view. A meta-analysis discovered no discernible link between psychopathy scores and leadership emergence and effectiveness (Landay et al., 2019). The intricacy intensifies within adolescent groups, where the factors that typically hinder individuals with high psychopathy scores in work settings, such as impulsivity, deviant behavior, and resistance to authority, might paradoxically construct an appealing antihero image (Jonason et al., 2012). Supporting this notion, Davis et al. (2022) identified a positive association between psychopathy and dating success among adolescents.

It is unlikely that individuals with psychopathic traits are particularly liked by their peers as they are probably not seen as friendly and cooperative (van der Linden et al., 2010). Self-reports indicate they form friendships with volatile others (Jonason & Schmitt, 2012), and employ distant or indirect communication when ending a friendship (Brewer et al., 2023). In the perceptions of others, they are predominantly viewed negatively (Rauthmann, 2012).

Forecasting the popularity and likeability of individuals possessing high levels of Machiavellianism presents a challenge due to the flexible and adaptable nature of their behavior. Previous research reveals a connection between Machiavellianism and a spectrum of both anti- and prosocial behaviors (e.g., Szabó et al., 2018). Anchored in the pursuit of personal success through diverse means, their behavior remains shaped by strategic objectives. For instance, Becker and O’Hair (2007) suggest that individuals with high levels of Machiavellianism exhibit altruistic behaviors when such actions aid in manipulating others’ perceptions of them. However, they tend to choose friends who are more vulnerable to exploitation (Jonason & Schmitt, 2012). In their friendships, they exhibit insincerity, demonstrate restricted self-disclosure, and may even display happiness in response to their friends’ misfortunes (Abell & Brewer, 2018; Brewer et al., 2014). They are largely seen negatively by others (Rauthmann, 2012).

Rogoza et al. (2021) investigated the simultaneous impact of the Dark Triad traits on relationship formation within a longitudinal and naturalistic setting. They collected self-report data on DT and obtained peer ratings from high-school classmates. Peers were asked to identify the individuals they liked the most in the classroom and those they perceived as leaders. The first question directly measured likeability, while the second question indirectly assessed popularity, or as argued by the authors, peer-rated agency. The study yielded complex relationships among the variables.

In the short term, psychopathy was negatively related to likeability, while Machiavellianism showed a positive relationship, and narcissism exhibited no significant association. Conversely, in the long term, only narcissism displayed a connection with likeability, acting as a negative predictor. This indicates that individuals with narcissistic traits were less favored by their peers over an extended time, in line with similar findings by Paulhus (1998).

In the short term, psychopathy was negatively related to leadership selection, while Machiavellianism showed a positive relationship, and narcissism exhibited no significant association. However, in the long term, narcissism emerged as the sole significant predictor of leadership selections. Specifically, individuals with narcissistic traits displayed a higher likelihood of being chosen as leaders, highlighting a peer-reported agency associated with narcissism. Closing this section, we would like to discuss the evolutionary psychological approach to DT. As mentioned earlier, some literature (de Vries et al., 2020) views the achievement of likeability and popularity as a solution to the evolutionary problem of belongingness. The evolutionary approach to DT has conceptualized these traits as condition-dependent adaptations to address life’s challenges within an unpredictable environment (Jonason et al., 2016). While directly testing this idea is almost impossible, it theoretically makes sense to consider DT as solutions for fulfilling belongingness needs through the attainment of social dominance, i.e., popularity.

Specifically, according to life history theory, individuals develop a life history shaped by their early experiences, which can be positioned along the slow-fast continuum (Belsky et al., 1991; Del Giudice et al., 2015). A fast life history can be characterized by an untrustful internal working model, utilitarian interpersonal relationships, early puberty, and sexual activity, among others. There is a close link between DT and a fast life history (Birkás et al., 2020; however, for an in-depth discussion about the complex relationships between DT and life history see Manson, 2020; McDonald et al., 2012). Many attributes of DT, such as impulsivity and entitlement, can be perceived as adaptive strategies developed in response to harsh and unpredictable environments (Jonason et al., 2016; McDonald et al., 2012).

Living a fast life is probably associated with the tendency to seek popularity—that is, dominance, visibility, and prestige. Essentially, a fast life history aligns with a ‘getting ahead’ motivation rather than a ‘getting along’ motivation. Adolescents in their turbulent and stressful development stage might take advantage of DT related to fast life history to achieve, often short-lived, popularity in peer groups which would be reflected in their higher popularity scores.

However, we do not posit a complete correspondence between DT and popularity. Specific DT attributes, such as aggression, can simultaneously promote and hinder popularity attainment, contingent on the type of aggression—proactive or reactive (Stoltz et al., 2016)

## The Present Study

The present study surveyed secondary school classes to explore the associations of self-reported DT with peer-rated likeability and popularity. The latter was measured through both direct and indirect means (peer-rated agency). Considering the conflicting possibilities and inconsistent findings in the limited existing research that has examined these matters, we are posing research questions. Nevertheless, some expectations can still be articulated, such as the anticipation that DT will exhibit a closer connection to popularity than to likeability.

*Questions related to likeability (RQ1):* What is the correlation between students’ self-reported DT and their peer-rated likeability? Do students with high self-reported Dark Triad traits receive favorable opinions and acceptance, and are they perceived as friendly and cooperative?

*Questions related to popularity (RQ2):* What is the correlation between students’ self-reported DT and their peer-rated popularity? Are students with high self-reported DT traits seen as popular among their peers and teachers? Additionally, are students with high-self reported DT seen as potential leaders among their peers and perceived to have successfully adapted to school life and possess great potential for success in life?

Importantly, the present study observed the associations between DT, likeability, and popularity in a highly naturalistic setting and with the employment of peer ratings. The literature reviewed above reveals that most of the relevant studies were conducted in less naturalistic settings and/or were based on self-report data.

The data, materials, and analysis syntax of the study are openly available at Szabó (2024).

## Method

### Sample

The study involved 184 secondary students (98 females and 86 males; *M*_AGE_ = 16.29, *SD* = 1.36) from nine classes enrolled in four-year secondary programs. Class size varied between 15 and 35 students. A post-hoc power analysis using G*Power (Faul et al., 2009) yielded acceptable power numbers (ranging between 0.52 and 0.99).

### Design and Procedure

Data were collected between September 2020 and January 2021 during the COVID-19 pandemic. Copies of the test battery were distributed among the students by their respective class teachers, as researchers were not authorized to enter the school site due to epidemiological reasons. To minimize the occurrence of missing cases, participants who were absent from school during their respective survey sessions were granted online access to the survey. Survey completion took approximately 45 minutes.

Before participating, each participant was informed about the study’s purpose and the relevant data protection provisions, particularly concerning the confidential handling of their data. Data collectors also ensured that all participants were at least 14 years of age. Following the provision of informed consent and demographic data, participants completed a self-report measure of the Dark Triad traits, followed by a sociometric inventory. Each participant submitted their response in a sealed envelope. Although collected data included participants’ names, we assured participants that their personal information would not be shared with third parties, including teachers, classmates, and parents.

Additionally, parental consent and institutional approval from the heads of the participating high schools were obtained before data collection. The study was conducted with ethical approval from Eötvös Loránd University.

### Measures

#### Dark Triad Personality Traits

We assessed DT using the 27-item Short Dark Triad (SD3; Jones & Paulhus, 2014), which was adapted to Hungarian by Szabó, Czibor et al. (2023). Each trait was measured by nine items: Machiavellianism (e.g., “It’s not wise to tell your secrets”; Cronbach’s α = 0.65), subclinical narcissism (e.g., “People see me as a natural leader”; Cronbach’s α = 0.76), and subclinical psychopathy (e.g., “People who mess with me always regret it”; Cronbach’s α = 0.71). Participants rated items on a 5-point scale ranging from “strongly disagree” to “strongly agree.” Composite scores were computed by calculating the mean of the corresponding items.

#### Likeability

Likeability was measured with six items focusing on intimate interpersonal relationships within the class: ‘Who is your best friend in the class?’; ‘Who would you first ask for help if you were in trouble?’; ‘With whom would you first share some interesting news?’; ‘Whom do you intend to stay in touch with after graduation?’; ‘Whom do you like the most in your class?’; ‘With whom would you first share a personal secret?’. Responses to these items were combined into a single composite variable labeled Likeability, which represented the sum of votes received by each participant across the six items.

#### Popularity

The measurement of popularity utilized two distinct methods. For the *direct* measure of popularity, participants were asked to identify the most popular members within their groups. We used two items to assess perceived popularity among peers (‘Who are the most popular among students in your class?’) and teachers (‘Who are the most popular students among teachers?’). This distinction enabled an exploration of potential variations in factors influencing popularity among students, as opposed to perceived popularity among authority figures. Importantly, it is crucial to clarify that both the perceived popularity among peers and teachers were evaluated through responses from peers. Teachers were not surveyed in this study.

*Indirect* measures of popularity assessed different aspects of agency, each captured by a single item: Leadership ability (‘Which of your classmates would be the best leader?’), Organizing skills (‘If your class teacher was unable to manage the class for an extended period, which of your classmates do you believe would be the most suitable replacement?’), Future success (‘Among your classmates, who do you believe will achieve the most success in life?’), and Adjustment to school life (‘Among your classmates, who appears most comfortable with school life?’).

Participants could select as many classmates as they wished for each question. To ensure data consistency across classes of varying sizes for each sociometric measure, the total number of votes received by each participant on each measure was divided by the total number of students in the participant’s class. Additional sociometric data on attractiveness and giftedness were collected but were not analyzed in the present data.

## Results

First, we investigated the correlations between DT and the sociometric variables (see Table 1).

**Table 1:** Spearman Correlations and Descriptive Statistics for DT Traits

Variable	Machiavellianism	Narcissism	Psychopathy
Likeability	.17*	.17*	.12†
Perceived popularity among peers	.17*	.33**	.16*
Perceived popularity among teachers	-.01	-.05	-.16*
Leadership ability	.12	.31**	.13†
Organizing skills	.06	.13	.03
Future success	.13†	.28**	.08
Adjustment to school life	-.07	.08	-.01
*M*	3.61	2.71	2.31
*SD*	0.60	0.71	0.68

*Note*. Correlation coefficients are Spearman’s rho.

†*p* < .10. *p < .05. **p < .01.

The Dark Triad traits exhibited weak, but statistically significant positive correlations with likeability. Narcissism displayed positive correlations with the number of votes related to perceived popularity among peers, perceived popularity among teachers, leadership, and potential for success in life. Machiavellianism was positively linked with perceived popularity among peers and exhibited a marginally significant correlation with perceived potential for success in life. Psychopathy displayed a positive correlation with perceived popularity among classmates while showing a negative correlation with perceived popularity among teachers.

Due to the relatively high frequency of zero values in each sociometric measure (ranging from 41.3% to 69%), a two-step analysis procedure, as proposed by Boulton and Williford (2018), was employed. For each sociometric measure, except likeability which possessed a continuous nature, a two-variable approach was adopted. This approach involved creating a binary variable (0 = no vote, 1 = one or more votes) and a continuous variable. In the continuous variable, zero values were excluded from subsequent analysis as missing data.

The binary variables were entered as outcome variables in uniform binary logistic regression models, using the DT as predictors and gender as a control variable. The continuous variables were included in linear regression models using the same predictor and control variables as the binary logistic models. We applied a Bonferroni-corrected *p*-value of .008 to determine the significance in the binary logistic regressions and .007 in the linear regressions. This correction involved dividing the threshold of .05 by the number of multiple comparisons.

In the binary logistic regressions, we report standardized beta weights (β), Wald statistics, Cox and Snell *R*^2^, and Nagelkerke *R*^2^. The results, presented in Table 2, indicated that narcissism was a significant predictor of perceived popularity among peers, perceived leadership ability, and perceived likelihood of future success. However, Machiavellianism and psychopathy did not emerge as significant predictors of the outcome variables.

**Table 2:** Results of Binary Logistic Regression on Popularity

	DV1: Perceived popularity among peers				DV2: Perceived popularity among teachers				DV3: Leadership ability			
Variable	β	*SE*	Wald	*p*	β	*SE*	Wald	*p*	β	*SE*	Wald	*p*
Mach	.39	.29	1.80	.18	.02	.27	.01	.94	.17	.30	.32	.58
Narc	.97	.27	13.26	<.001	-.03	.23	.01	.91	.80	.26	9.13	.003
Psych	.07	.27	.06	.80	-.20	.25	.61	.44	.24	.27	.77	.38
Gender	-.72	.33	4.70	.03	-.51	.31	2.71	.10	-.02	.34	.01	.94
Cox and Snell *R*^2^	.12				.02				.08			
Nagelkerke *R*^2^	.16				.03				.12			
	DV4: Organizing skills				DV5: Future success				DV6: Adjustment to school life			
Variable	β	*SE*	Wald	*p*	β	*SE*	Wald	*p*	β	*SE*	Wald	*p*
Mach	.23	.28	.64	.43	.16	.28	.34	.56	-.52	.29	3.10	.08
Narc	.19	.24	.64	.43	.64	.25	6.55	.01	.23	.25	.85	.36
Psych	.07	.26	.08	.77	-.02	.26	.00	.95	.13	.27	.23	.63
Gender	-.28	.32	.77	.38	-.25	.32	.62	.43	.52	.33	2.52	.11
Cox and Snell *R*^2^	.01				.05				.04			
Nagelkerke *R*^2^	.02				.07				.05			

*Note.* Mach = Machiavellianism. Narc = Subclinical narcissism. Psych = Subclinical psychopathy.

Table 3 presents the results of the linear regression analyses. Narcissism emerged as a significant positive predictor of perceived leadership ability and perceived organizational skills. Individuals exhibiting narcissistic traits were also more likely to be perceived as destined for future success. Additionally, several other associations reached the conventional significance threshold (*p* < .05) but did not achieve Bonferroni-corrected significance (*p* < .07). For instance, narcissism was a positive predictor of perceived popularity among peers (*p* = .05) and perceived popularity among teachers (*p* = .01), while psychopathy was a negative predictor of perceived popularity among teachers (*p* = .02). Psychopathy was also a negative predictor of perceived leadership abilities (*p* = .02) and the perceived ability to adapt to school life (*p* = .04). Machiavellianism was a positive predictor of the perceived ability to fit into school life (*p* = .02).

**Table 3:** Regression Analyses on Likeability and Popularity

	DV1: Perceived popularity among peers				DV2: Perceived popularity among teachers			
Variable	β	*SE*	β *95% CI*	*p*	β	*SE*	β *95% CI*	*p*
Mach	.00	.02	-.04, .05	.91	.04	.03	-.01, .09	.12
Narc	.04	.02	.00, .08	.05	.06	.02	.02, .10	.01
Psych	.01	.02	-.04, .05	.76	-.06	.02	-.10, -.01	.02
Gender	-.04	.03	-.02, .09	.19	-.03	.03	-.03, .09	.29
	Model summary: *R*^2^ = .06, *F*(4, 95) = 1.48, *p* = .22				Model summary *R*^2^ = .13, *F*(4, 81) = 3.11, *p* = .02			
	DV3: Leadership abilities				DV4: Organizing skills			
Variable	β	*SE*	β *95% CI*	*p*	β	*SE*	β *95% CI*	*p*
Mach	.03	.03	-.03, .09	.38	-.02	.03	-.08, .04	.42
Narc	.09	.03	.04, .14	.001	.10	.03	.05, .15	<.001
Psych	-.06	.02	-.11, -.01	.02	-.05	.03	-.10, .01	.10
Gender	-.09	.03	.03, .15	.01	-.06	.03	.00, .13	.05
Model summary: *R*^2^ = .30, *F*(4, 52) = 5.43, *p* = .001					Model summary *R*^2^ = .24, *F*(4, 59) = 4.61, *p* = .003			
	DV5: Future success				DV6: Adjustment to school life			
Variable	β	*SE*	β *95% CI*	*p*	β	*SE*	β *95% CI*	*p*
Mach	.00	.02	-.04, .03	.90	.05	.02	.01, .09	.02
Narc	.05	.02	.02, .08	.001	.01	.02	-.03, .04	.69
Psych	-.01	.02	-.04, .02	.53	-.04	.02	-.07, .00	.04
Gender	-.06	.02	.02, .10	.01	-.03	.02	-.01, .07	.15
Model summary: *R*^2^ = .13, *F*(4, 103) = 3.94, *p* = .005					Model summary *R*^2^ = .13, *F*(4, 56) = 2.05, *p* = .10			
	DV7: Likeability							
Variable	β	*SE*	β *95% CI*	*p*
Mach	.01	.01	.00, .03	.10				
Narc	.01	.01	.00, .03	.05				
Psych	.01	.01	.00, .03	.14				
Gender	-.03	.01	.02, .05	< .001				
Model summary: *R*^2^ = .12, *F*(4, 179) = 6.29, *p <* .001								

*Note.* Mach = Machiavellianism. Narc = Subclinical narcissism. Psych = Subclinical psychopathy.

## Discussion

The present study investigated the associations between DT, likeability, and popularity in a naturalistic setting, employing both self-rated and other-rated data.

The most consistent results were obtained for narcissism. Specifically, individuals with higher self-reported narcissism were perceived by their classmates as popular and likable. They were also seen as possessing strong leadership and organizational abilities. Furthermore, they were more frequently identified as having substantial potential for post-secondary school success compared to individuals with lower narcissistic traits. The predictive impact of psychopathy and Machiavellianism in terms of likeability and popularity was found to be minimal. Some indications emerged that individuals with high psychopathy and Machiavellianism scores are marginally more liked by their peers and slightly more popular compared to other students. Furthermore, individuals with elevated Machiavellianism scores exhibit adeptness at effectively adapting to their current social environment. However, when considering the broader context, our findings surprisingly suggest that neither Machiavellianism nor psychopathy is correlated with social acceptance within adolescent groups.

Due to the limitations of our study design, our results are primarily observational, and the explanations we provide are inherently speculative. Nevertheless, we consider these observations valuable as they contribute to our understanding of two important questions in the literature: (1) the factors that account for the likeability and popularity of members within adolescent groups, and (2) the perception of individuals with Dark Triad traits by their closely associated peers.

The likeability and popularity associated with narcissism could potentially demonstrate the accuracy of self-perceptions among individuals with elevated narcissism. Future studies should investigate in greater detail whether individuals with high narcissism levels are indeed more likable and popular compared to those with lower narcissistic tendencies. Alternatively, these results are only illustrations of the transient effectiveness of narcissistic individuals’ self-perception strategies, rather than unequivocally indicating their genuine acceptance among peers. Rogoza et al. (2021) have demonstrated dynamic shifts in the likeability and perceived leadership abilities of individuals with elevated narcissism over the long term.

Nevertheless, it is intriguing to compare our findings with those of Rogoza et al. (2021). In their study, they initially found no significant relationship between narcissism and likeability or perceived leadership ability. However, in the long term, narcissism exhibited a negative correlation with likeability and a positive correlation with perceived leadership ability. This suggests that individuals with higher narcissism scores were seemingly regarded by their peers as more capable leaders in both studies. Their ‘born leader’ attitude could either be grounded in realistic self-assessments or function as a self-fulfilling prophecy. However, the extent to which peers can form positive likeability perceptions of individuals with elevated narcissism scores remains an open question.

Rogoza et al. (2021), akin to our study, employed the SD3 to assess Dark Triad traits. This assessment predominantly captures the agentic aspects of narcissism. Future studies might consider utilizing measures that encompass additional aspects of narcissism such as vulnerability and antagonism (Crowe et al., 2019).

The null effects associated with Machiavellianism and psychopathy may be attributed to the simultaneous influence of Machiavellianism and narcissism characteristics, which contribute to both promoting popularity and eliciting rejection. The inclination of individuals with high psychopathy scores to rebel against authority, take risks, and engage in instrumental aggression may facilitate their attainment of social dominance. However, simultaneously, their harassment of peers, hostile aggression, and self-centered, exploitative behaviors may easily overshadow any popularity they may have gained.

Likewise, the adaptive behavioral strategies characteristic of Machiavellians could function both in favor of and against the attainment of popularity. Individuals with high Machiavellianism scores tend to favor partners they can exploit (Jonason & Schmitt, 2012) and they display self-serving behaviors even within collaborative groups (Bereczkei et al., 2015). This may lead to greater success in short-term relationships, yet it can also lead to conflicting evaluations within communities where members interact daily over an extended period, such as a high school classroom. While speculative, our analysis leads us to conclude that the advantageous and disadvantageous facets of psychopathy and Machiavellianism, in terms of fostering popularity, may counterbalance each other, resulting in null effects.

Based on these findings, it would certainly be a far-fetched conclusion to suggest that Machiavellianism and psychopathy are evolutionary solutions for the adaptive developmental problem of peer belongingness needs (de Vries et al., 2020). On the other hand, narcissism was positively associated with both likeability and popularity. It seems that narcissistic traits such as self-confidence, grandiosity, exhibitionism, and a ‘born leader’ attitude may appeal to members of an adolescent peer group (Jonason et al., 2012). Thus, the obtained findings for narcissism are consistent with previously proposed evolutionary conceptualizations (e.g., Jonason et al., 2016).

### Limitations and Future Studies

Several limitations should be acknowledged. The cross-sectional, non-longitudinal design of the study precludes analyzing the DT’s associations with long-term social success. Future research could adopt a longitudinal design to explore temporal changes in the relationship between DT and the attainment of likeability and popularity.

Another limitation is the relatively small sample size due to the then-active coronavirus pandemic. This implies that any observed effects should be interpreted with caution. However, we believe that the richness of our data compensates for this limitation. For instance, we utilized 12 sociometric items to access likeability and popularity, while Rogoza et al. (2021), in a comparable sociometric investigation, only employed two items. Our sample size is comparable to those used in previous studies investigating the likeability and popularity of individuals with high DT (see e.g., Jauk et al., 2016; Rauthmann, 2012; Rogoza et al., 2021).

Additional limitations pertain to the instruments employed in this study. The SD3, though practical, primarily focuses on the agentic aspect of narcissism, neglecting facets like antagonism and vulnerability that could impact social acceptance. Also, the Machiavellianism subscale’s Cronbach’s alpha fell below the traditional cutoff (Nunnally, 1978). Future studies should employ more reliable, age-specific measures and expand assessment scope by evaluating each DT trait separately using comprehensive measures. Future research could also explore additional dark traits, such as everyday sadism (Buckels et al., 2013).

### Conclusion

The primary outcome of our research indicates a clear correlation between narcissism and both likeability and popularity. Psychopathy and Machiavellianism, on the other hand, exhibit minimal associations with factors measuring social acceptance.

Understanding the determinants of peer acceptance during adolescence holds both academic intrigue and practical significance. The existing literature suggests that individuals who do not achieve either pathway of peer acceptance -meaning they are neither popular nor liked- experience negative consequences in the short and long term, impacting their well-being and potentially their academic performance. Consequently, any insights that deepen our understanding of peer acceptance during adolescence can equip educators with valuable tools to support a broader range of students in gaining peer acceptance.

Our findings could also foster the development of empathy and perspective-taking among professionals working with young individuals. These findings show that personality traits conventionally labeled as dark, such as narcissism, can also be seen as solutions to adaptive developmental challenges. Despite the evident drawbacks of inflated self-esteem, entitlement, and exploitation of others, these behaviors can also be interpreted as adolescents’ attempts to establish dominance, prestige, visibility, and, consequently, a sense of belongingness.

## Data Availability

The data, materials, and analysis syntax of the study are openly available at Szabó (2024).
